# Laser-Induced Chirality of Plasmonic Nanoparticles Embedded in Porous Matrix

**DOI:** 10.3390/nano13101634

**Published:** 2023-05-13

**Authors:** Anastasiia A. Sapunova, Yulia I. Yandybaeva, Roman A. Zakoldaev, Alexandra V. Afanasjeva, Olga V. Andreeva, Igor A. Gladskikh, Tigran A. Vartanyan, Daler R. Dadadzhanov

**Affiliations:** 1 International Research and Education Center for Physics of Nanostructures, ITMO University, 49 Kronverksky pr., St. Petersburg 197101, Russia; spnvnastja@gmail.com (A.A.S.); afanasjeva.sasha2011@yandex.ru (A.V.A.); 138020@mail.ru (I.A.G.); 2 Institute of Laser Technology, ITMO University, 49 Kronverksky pr., St. Petersburg 197101, Russia; yulia.yandybaeva@gmail.com (Y.I.Y.); zakoldaev@gmail.com (R.A.Z.); 3 Research and Educational Center for Photonics and Optoinformatics, ITMO University, 49 Kronverksky pr., St. Petersburg 197101, Russia; olga_andreeva@mail.ru

**Keywords:** chirality, chiral plasmonics, circular dichroism, plasmonic nanoparticles, gold, LSPR, nanoporous, optical activity, 2D materials, 3D materials

## Abstract

Chiral plasmonic nanostructures have emerged as promising objects for numerous applications in nanophotonics, optoelectronics, biosensing, chemistry, and pharmacy. Here, we propose a novel method to induce strong chirality in achiral ensembles of gold nanoparticles via irradiation with circularly-polarized light of a picosecond Nd:YAG laser. Embedding of gold nanoparticles into a nanoporous silicate matrix leads to the formation of a racemic mixture of metal nanoparticles of different chirality that is enhanced by highly asymmetric dielectric environment of the nanoporous matrix. Then, illumination with intense circularly-polarized light selectively modifies the particles with the chirality defined by the handedness of the laser light, while their “enantiomers” survive the laser action almost unaffected. This novel modification of the spectral hole burning technique leads to the formation of an ensemble of plasmonic metal nanoparticles that demonstrates circular dichroism up to 100 mdeg. An unforeseen peculiarity of the chiral nanostructures obtained in this way is that 2D and 3D nanostructures contribute almost equally to the observed circular dichroism signals. Thus, the circular dichroism is neither even nor odd under reversal of direction of light propagation. These findings will help guide the development of a passive optical modulator and nanoplatform for enhanced chiral sensing and catalysis.

## 1. Introduction

No one doubts the fact that designing nanoparticles with one symmetry or another is an extremely difficult task. Strictly speaking, the nanoparticles resulting from any textcolorbluefabrication method do not have any symmetries, that is, they are asymmetric. Asymmetrical objects are always chiral, which means they cannot be aligned with their mirror image. Chiral nanostructures have many properties that are interesting for different applications: they can rotate the plane of light polarization and they can interact selectively with other chiral objects [1]. In particular, they absorb light with the left-handed (LHCP) and right-handed circular polarization (RHCP) differently. This effect, termed as circular dichroism (CD), allows studying chiral structures with CD spectroscopy. In pharmaceuticals, medicine, and chemical technologies, the study of chirality in molecules such as proteins [2], peptides [3], nucleotides [4], and DNA/RNA [5] is particularly relevant. Unfortunately, the CD signal of these molecules is very weak. To recognize it, either a larger amount of the detectable substance or auxiliary pathways is required. Advanced nanostructures can be used to overcome this difficulties.

Recently, there has been active research on chirality properties of advanced nanostructures such as carbon nanostructures [6,7], semiconductor nanocrystals [8], and plasmonic nanoparticles [9]. Chirality of the listed nanostructures is several times higher than that of natural molecules, making them indispensable assistants in research. Advanced chiral nanostructures that repeat the properties of natural chiral molecules can be fabricated in a certain way. The interaction between chiral nanostructures and natural chiral objects enhances the CD signal of the latter ones, making them observable in a wide range of the spectrum. Chiral nanoparticles are widely used in rapidly developing fields such as chiral sensing [10,11], biomolecular recognition [12], cell imaging [13], cytotoxicity control [14], design of circularly polarized light sources, asymmetric catalysis [15], and circular polarized luminescence (CPL) [16]. A variety of potential applications motivated the development of different ways of inducing chirality and synthesis chiral nanoparticles, especially in plasmonics, since plasmonic materials can effectively enhance weak signals due to their strong light–matter interaction [17].

The growing interest in chiral plasmonics leads to simplifying the approaches of fabricating chiral plasmonic nanoparticles (NPs). The objective of designing chiral structures is quite difficult, since the nanostructures must be both reproducible and efficient in their use in further experiments. The most popular methods for fabricating chiral plasmonic NPs are lithography, ion beam etching, direct laser writing, and chemical synthesis based on natural chiral molecules [9]. Nanosphere lithography, which involves a layer of inert nanospheres as a mask, can be used for the chiral nanoparticles fabrication [18] as well. The main disadvantage of this method is its high cost, which prevents mass production for sensing applications. Moreover, lithographic methods include a procedure for removing nanoparticles by strong etchants, which greatly affects the result [19].

During the focused ion etching procedure, chiral plasmonic NPs can be produced by removing the material from the surface under the influence of a high-energy ion beam with a gas exchange precursor in a vacuum environment [20]. Using lenses, the beam is focused on the material. Along with lithography, fabricating plasmonic NPs with a focused ion beam has its bright advantages, for example, high reproducibility and control of the nanoparticles shape and size. However, there are also the same critical drawbacks—complex geometries are possible by using automatic design and production systems, which significantly increases the cost of this method and restricts mass-production use.

In constrast to lithography and ion beam etching, chiral plasmonic NPs can be obtained by self-assembled method using chiral molecules—for example, DNA—as a matrix [21]. Otherwise, this method is referred to as the method of transferring molecular chirality to nanomaterials. The main advantage of this method is that nanostructures can be synthesized with arbitrary shapes and sizes, while in the case of lithography, a homogeneous array of structures is obtained. The main disadvantage of this method is the limited availability of chiral matrices; otherwise, in the absence of a matrix, the chirality of nanoparticles tends to disappear. In turn, direct laser writing allows fabricating both 2D and 3D plasmonic nanostructures by employing two-photon absorption in photoresist and subsequent procedures such as metal doping, evaporation, or electroplating. When the laser is exposed to the photoresist material, thermal heating occurs at the site of exposure leading to formation of “islands” in the focus. Owing to the filling the voids with plasmonic materials, chiral nanoparticles can be fabricated [22,23].

It is to be stressed that there are two types of chiral objects that lead to similar, although not identical, CD effects, namely 3D and 2D chiral objects. The 3D chiral objects are chiral in the rigorous sense, as they cannot be brought into coincidence with their mirror images by any movement in the 3D space. The sign of CD signals produced by 3D chiral objects is independent of their orientation relative to the direction of light propagation. On the other hand, the 2D chiral objects cannot be brought into coincidence with their mirror images by any movement restricted to the plane where they reside. If 3D rotations are allowed, such objects may be brought into coincidence with their mirror images. Hence, they are not chiral in the rigorous sense. Nevertheless, they do differ in the absorption cross sections for LHCP and RHCP light. The only departure from the properties of the 3D chiral objects is that the sign of the CD of 2D chiral objects is defined by the direction of light propagation.

In this paper, we describe a novel method to induce chirality in achiral ensembles of gold NPs using the pulsed laser irradiation with circular polarization. We employ the method of spectral hole burning [24,25,26,27]. The essence of the method is that irradiation of plasmonic NPs with a resonant wavelength causes the particles to melt, change shape, and migrate. The process leads to the formation of a dip in the absorption spectrum at the irradiation wavelength [27,28,29]. Contrary to all existing methods of fabrication of chiral plasmonic NPs, we propose here for the first time an effective method of inducing chirality in gold NPs embedded in the nanoporous silicate matrix (NPSM) using the spectral hole burning technique. The idea of the method is based on the very probable assumption that the synthesized nanoparticles are not only not ideal spheres, but in general, they do not have any symmetry in the strict sense, that is, they are asymmetric. Since asymmetric objects are always chiral, an ensemble of such particles can be expected to exhibit circular dichroism, provided that the numbers of right and left “enantiomers” are different. Of course, before laser irradiation, the whole ensemble of nanoparticles is a “racemic mixture” of objects with different chirality. Thus, the CD signals of unilluminated samples remain very near to zero. The terms enantiomer and racemic mixture are used here in a broader sense. Indeed, as all nanoparticles are different, it is not possible to find pairs of particles that are exact mirror images of each other. Despite this, the random nature of deviations of the particle shape from any symmetry guarantees the absence of circular dichroism in an unirradiated ensemble of nanoparticles. Then, illumination of the NPs with the intense circular polarized light can selectively burn, or at least predominantly modify, particles with chirality defined by the handedness of the laser light, while their “enantiomers” will survive the laser action almost unaffected. In what follows, we show that this method makes it possible to obtain strong CD signals at low concentrations of gold NPs. Of special interest is the difference in CD signals measured from the opposite sides of the sample and the possibility to diminish the CD signals by further irradiation with laser light of opposite handedness.

## 2. Materials and Methods

### 2.1. Chemicals

Cetyltrimethylammonium bromide (CTAB, ≥99%), hydrogen tetrachloroaurate (III) trihydrate (HAuCl${}_{4}\cdot$3H${}_{2}$O, ≥99.99% purity-trace metal basis), sodium borohydride (NaBH${}_{4}$, powder, ≥98.0%), and sodium hydroxide (NaOH, ≥98.0%) were purchased from Sigma Aldrich. Deionized water (resistivity 18.2 MΩ·cm) was used for synthesis of gold NPs.

### 2.2. Synthesis of Gold NPs

**Figure A1:**
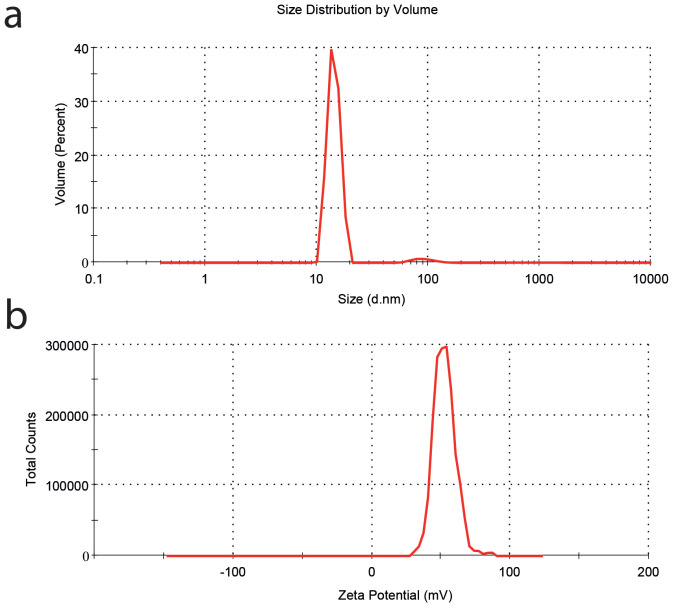
(**a**) DLS size distribution and (**b**) ζ-potential histogram of gold nanoparticles obtained in deonized water.

**Figure A2:**
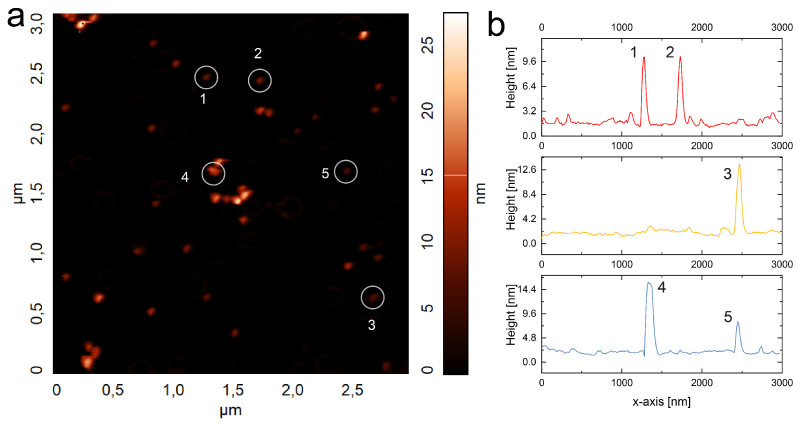
(**a**) 2D images of the gold nanoparticles obtained by atomic force microscope and images with (**b**) horizontal section across several nanoparticles. The digits in (**b**) correspond to region of interest with the single gold nanoparticle in (**a**).

A seed solution was prepared according to procedure described in [30]. Briefly, we prepared a solution containing 0.2 mL of 0.025 M HAuCl${}_{4}\cdot$3H${}_{2}$O and 9.5 mL of 0.10 M CTAB. Next, ice-cold, freshly prepared 0.46 mL of 0.010 M NaBH${}_{4}$ in 0.010 M of NaOH was quickly added to the stirred gold solution. Immediately, the solution turned from yellow to brown. After that, the solution was stirred for 10 min. Then, it was kept unstirred at room temperature for 2 h. As shown in the DLS measurement of the gold nanoparticles in Figure A1, the average size of nanoparticles was 14.3 nm. The analysis of the ζ-potential distribution of gold NPs in deionized water at 25 °C and a pH of 6.48 indicates the average ζ-potential value as 52.3 mV (Figure A2).

### 2.3. Incorporation of Gold NPs into NPSM

**Figure 1:**
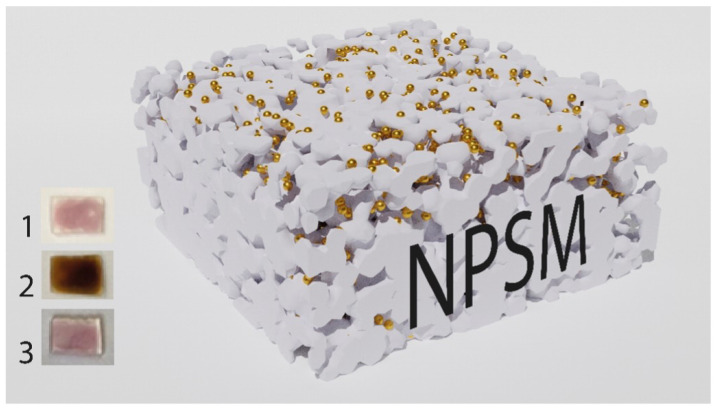
3D model of the composite structure with a racemic ensemble of gold NPs embedded into the NPSM. The images under numbers 1, 2, and 3 represent the Au/NPSM composite structures after annealing at 50 °C, 200 °C, and 500 °C, correspondently.

Nanoporous silicate matrix (NPSM) was fabricated according to the procedure described in [31]. This matrix has an average pore size of 17 nm, high porosity (50–54%), and refractive index of 1.22 [32]. The matrix consists of 91.4% SiO${}_{2}$ and impurities: 7.4% B${}_{2}$O${}_{3}$, 1.2% Na${}_{2}$O. To remove contaminations after fabrication, NPSM was annealed at 500 °C in the furnace for an hour. Then, the colloidal solution of gold NPs with the concentration of 0.5 mM dispersed in deionized water was deposited on the surface of NPSM by the drop-casting method. Herein, we refer the deposition facet of gold NPs as the nanoparticle side and the opposite one as the backside (Figure 1).

**Figure A3:**
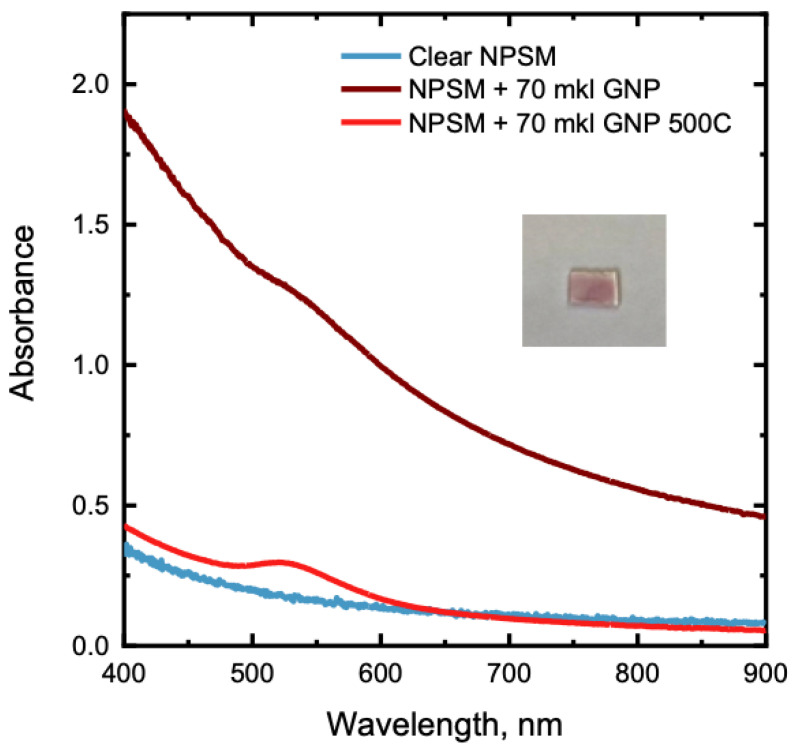
Absorption spectra of samples of blank NPSM and NPSM with gold NPs before and after high-temperature annealing.

The nanocomposite structure was subsequently dried on an electric furnace heated to 50 °C for an hour to evaporate water. The annealing of NPSM with gold NPs (Au/NPSM) was repeated until pronounced peak of plasmon resonance appeared as is shown in Figure A3 (brown curve). Since the initial colloidal solution of gold NPs in water contains excess CTAB molecules, the NPSM with gold NPs was annealed in a muffle furnace for an hour at the temperature of CTAB decomposition, which is 500 °C. Thus, the residual CTAB in the NPSM was thoroughly thermally removed, which no longer affects the structure, which in turn affects the circular dichroism spectra of gold NPs in NPSM. Finally, this procedure resulted in a more transparent structure of the nanocomposite, as shown in the third image in Figure 1 and the optical density spectrum in Figure A3 (red curve).

### 2.4. Laser Modification of Gold NPs in the Porous Matrix

**Figure 2:**
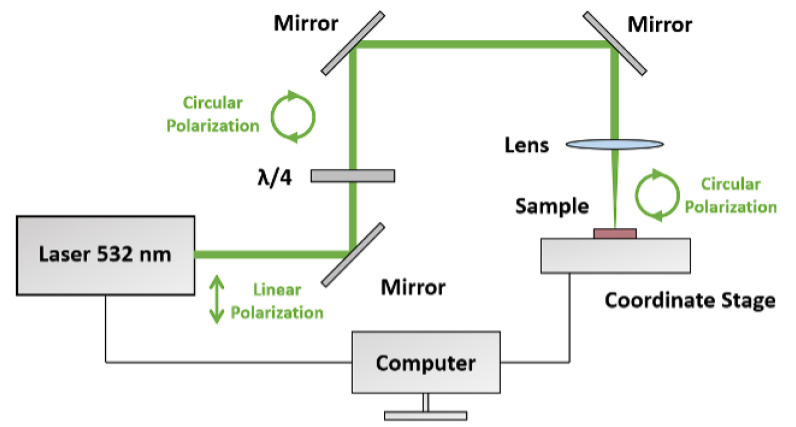
Scheme of irradiation of a racemic ensemble of gold NPs in the NPSM by the second harmonic of the picosecond Nd:YAG laser. The linearly polarized laser light was transformed to the right-handed circularly polarized light by a quarter-wave plate and focused on the surface of the gold NPs/NPSM composite structure with a lens.

Chirality was induced in a racemic ensemble of gold NPs inside the NPSM by illumination with circularly polarized light. The scheme of the experimental setup is shown in Figure 2. The linearly polarized second harmonic (λ = 532 nm) light of the picosecond Nd:YAG laser (EXPLA PL2143) was converted into right-handed circularly polarized light with the aid of a quarter-wave plate (OPTICS PROVIDER, St. Petersburg, Russia) rotated by −45${}^{\circ }$ with respect to the laser beam polarization axis, as shown in Figure 2. The laser beam was focused onto the nanoparticle side of the NPSM using a convex lens. The diameter of laser beam spot during the scanning of the Au/NPSM was 500 µm. The laser pulse duration was 30 ps, the repetition rate was 10 Hz, and the laser pulse energy was set to 45 µJ. To enlarge the laser modified area, the laser beam was scanned over the NPSM surface with the speed of 2.5 mm/s. To further investigate the origin and nature of the laser-induced CD in gold, the sample was re-irradiated with the right-handed circularly polarized light from the backside with the scanning speed of 0.5 mm/s.

### 2.5. Characterization

Circular dichroism spectra were obtained by using the circular dichroism spectrophotometer J-1500 (Jasco, Tokyo, Japan). Absorption spectra of colloidal gold NPs and gold NPs inside of the NPSM were measured using the photonic multichannel analyzer PMA-12 (Hamamatsu Photonics Co., Ltd., Shizuoka, Japan). Hydrodynamic size and ζ-potential of the colloidal gold NPs were determined using the Malvern Zetasizer Nano ZS system (Malvern Instruments Ltd., Malvern, UK). A surface morphology study was performed by atomic force microscopy (AFM) (NT-MDT SPM–Nova, Moscow, Russia).

## 3. Results and Discussion

**Figure 3:**
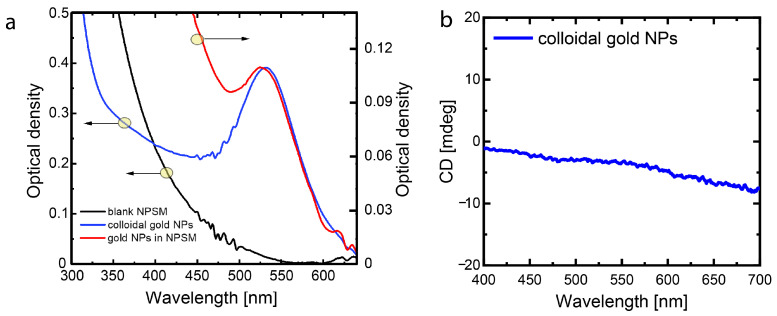
(**a**) Optical density spectra of the blank NPSM (black curve), colloidal gold NPs (blue curve), and NPSM with gold NPs (red curve). (**b**) CD spectrum of colloidal gold NPs in a 1 cm thick quartz cuvette.

Optical density spectra of the blank NPSM, colloidal solution of gold NPs, and gold NPs/NPSM composite structure after annealing are shown in Figure 3a. The bare NPSM is not fully transparent at the wavelengths smaller than 550 nm due to the growing light scattering in the porous structure at smaller wavelengths. As it was expected, the incorporation of gold NPs into the NPSM led to the blue-wavelength shift of the localized surface plasmon resonance since the effective refractive index of the NPSM (n = 1.22 [32] is lower than that of water (n = 1.33). Indeed, Figure 3a demonstrates that the peak of absorption of gold NPs inside the NPSM (gold NPs/NPSM composite structure) is shifted by 7 nm with respect to that of the colloidal gold NPs. Reshaping of gold NPs due to annealing after their incorporation into the NPSM can also contribute slightly to the blue-shift of the gold NPs’ localized surface plasmon resonance.

**Figure 4:**
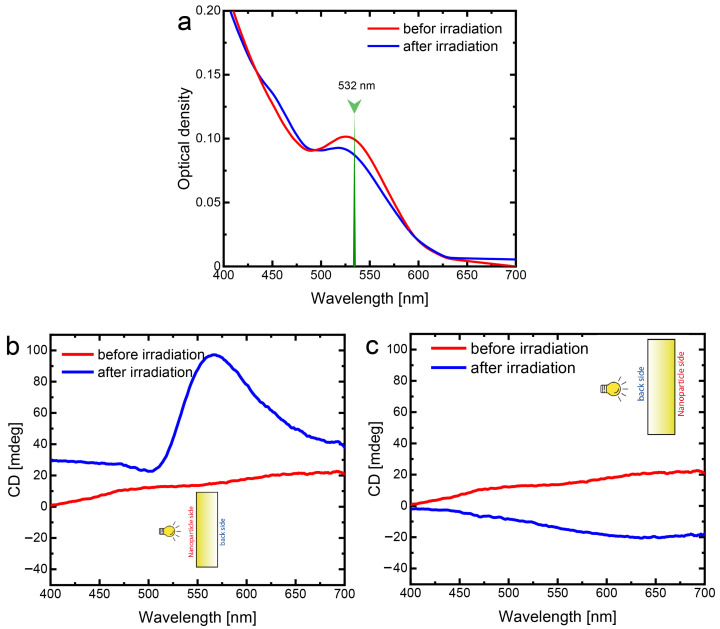
(**a**) Optical density spectra of the Au/NPSM composite structure before (red curve) and after (blue curve) irradiation by RHCP laser light at the wavelength of λ = 532 nm; (**b**) CD spectra measured from the nanoparticle side—the side of NPSM on which the gold NPs were deposited and from which they were irradiated by the RHCP laser light—before (red curve) and after (blue curve) irradiation by RHCP; (**c**) CD spectra of the same sample (irradiated from the nanoparticle side) but measured from the backside before (red curve) and after (blue curve) irradiation by RHCP. The insets in (**b**,**c**) demonstrate the position of the probe light source with respect to the NPs/NPSM sample in course of CD spectral measurements.

Negligibly small values of CD presented in Figure 3b testify that the colloidal gold NPs used for the further studies form a racemic mixture. After the embedding of these gold NPs inside the porous matrix their CD remains very small, as it may be clearly seen in Figure 4b,c (red curves). The relatively small variations of the CD signal from 0 to 20 mdeg can be attributed to the anisotropy of the NPSM. To observe the maximum effect of the laser irradiation, we employ the second harmonic of Nd:YAG laser, the wavelength of which (532 nm) is close to the absorption maximum of the plasmon resonance of gold NPs. Optical density (Figure 4a) and CD spectra (Figure 4b,c) indicate the Au/NPSM composite structure before and after irradiation by the right-handed circularly polarized light of the Nd:YAG laser. The decrease in the optical density after laser irradiation is an apparent consequence of the ordinary spectral hole burning due to the ablation of gold NPs. The novel and most interesting features were observed in the CD spectra that emerged after the laser irradiation. The CD spectra appeared to be different when measured from different sides of the Au/NPSM composite structure. When the probe light falls on the sample from the nanoparticle side—the side from which the sample was irradiated by the RHCP laser light (Figure 4b, blue curve)—CD values grow up to 100 mdeg in the narrow range from 500 to 650 nm. This is a major change compared to the vague variation in the whole visible range not exceeding 20 mdeg observed for the gold NPs/NPSM before laser treatment (Figure 4b, red curve).

**Figure A4:**
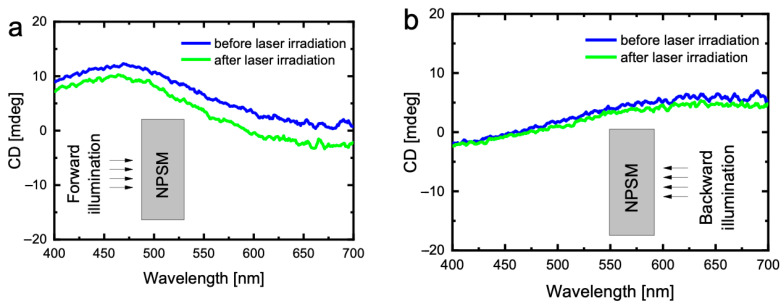
CD spectra of the blank NPSM measured from the nanoparticle side (**a**) and from the back side (**b**). Blue and green curves designate the CD of blank NPSM before and after laser irradiation, respectively.

On the other hand, the CD spectrum of the laser treated gold NPs/NPSM composite structure measured from the back side is much smaller and has the opposite sign as compared with that measured from the nanoparticles side. This may be clearly seen by comparison blue curves in Figure 4b,c. It is important to note that the CD spectrum of the gold NPs/NPSM composite structure before laser irradiation is independent of the side from which the measurement is performed. To elucidate the lack of effect from laser irradiation and side of light illumination, additional studies were performed. The intensity of CD signal of the laser irradiated NPSM is smaller than 10 mdeg as shown in Figure A4.

**Figure 5:**
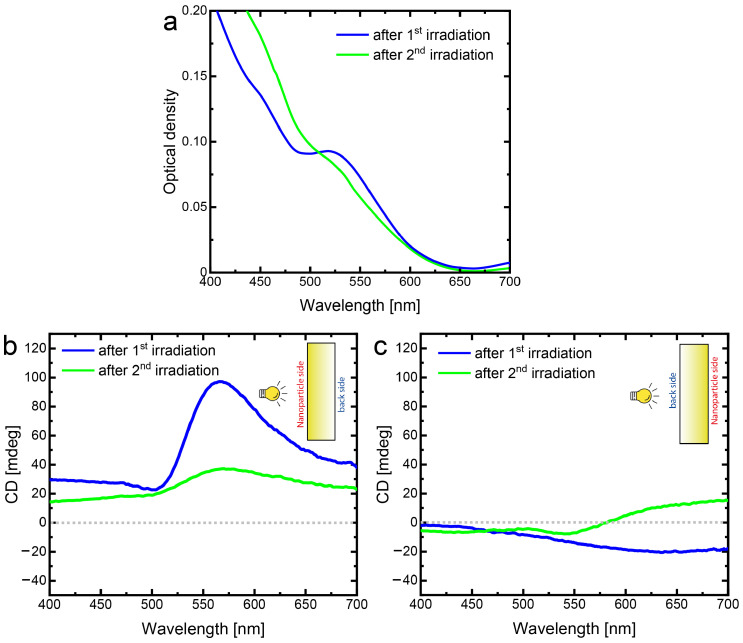
(**a**) Optical density spectra after the first (blue curve) and second (green curve) laser irradiations (532 nm, RHCP); CD spectra measured from (**b**) the frontside and (**c**) the backside of NPSM with gold NPs (blue curve correspond to the first irradiation, while the green curve corresponds to the second irradiation). Both irradiations were made with the RHCP light. The first irradiation was performed from the nanoparticle side, while the second irradiation was performed from the back side.

Figure 5 presents the optical density and CD spectra of gold NPs/NPSM after the second laser irradiation that was performed from the backside. As expected, the laser irradiation has led to further reduction of the gold NPs/NPSM composite structure absorption at the wavelengths around the laser wavelength of 532 nm due to the ablation of gold NPs. Thus, the laser irradiation from the back side enhanced the effect of the irradiation from the nanoparticle side.

Contrary to that, the magnitude of the CD signal induced by the first laser irradiation is largely reduced by the second laser irradiation. For comparison, Figure 5b plots the CD signals obtained after the first laser irradiation (blue curve) and after the second laser irradiation (green curve), both measured from the nanoparticle side. Evidently, after the second laser irradiations, the CD signal decreased more than two times. The CD signals measured from the backside are shown in Figure 5c. In this case, the changes, although present, are much less pronounced. The interpretation of the observations described above is premised on the generalized ideas of the hole burning technique. Gold nanoparticles incorporated into nanoporous silicate matrix are different in size and shape. Hence, they form an inhomogeneously broadened plasmon band in the spectral domain and a racemic mixture of different chiral species with negligible CD. Laser irradiation selectively affects nanoparticles whose plasmon resonance is closest to the wavelength of laser radiation, thus forming a spectral hole. In addition, irradiation with RHCL disrupts the balance between the nanoparticles of different shapes. The depth of the spectral hole for the right-hand circular polarized probe light is deeper than that for the left-hand circular polarized probe light. Thus, laser irradiation with RHCL induces CD in the spectral range of the burnt hole. The sort of the chirality was learnt from the results of CD measurement performed from the opposite side of the sample. According to the general considerations, the sign of the CD remains the same in the case of 3D chiral nanoparticles and changes its sign in the case of 2D chiral nanoparticles [33,34]. Thus, the CD measured from the side from which the sample was irradiated (in our case, the nanoparticles side) is the sum of the contributions of 3D and 2D chiral nanoparticles, while the CD measured from the opposite side (in our case, the back side) is the difference of contributions of 3D and 2D chiral nanoparticles. As in the last case, the measured CD is much smaller than in the former and has an opposite sign, one can conclude that 3D and 2D chiral nanoparticle contributions are almost equal with slight prevalence of the 2D contribution. Additional confirmations of the proposed interpretation of the experimental results come from the results obtained when the sample already irradiated from nanoparticle side was irradiated from the back side. Besides deepening of the spectral hole, laser irradiation from the back side diminishes the CD. Such a behavior is in accord with the expectations because in this case, laser irradiation predominantly affects nanoparticles of different shapes and partially restores the balance disrupted by the first irradiation.

## 4. Conclusions

In this work, a novel modification of the spectral hole burning technique was employed to induce strong circular dichroism in an ensemble of gold nanoparticles embedded in nanoporous silicate matrix. After irradiation with the picosecond pulses of right-handed circularly-polarized laser light at the wavelength of 532 nm, the nanoparticle ensemble inside NPSM demonstrates circular dichroism up to 100 mdeg. Circular dichroism of the plasmonic nanoparticles ensembles can be controlled by varying the irradiation parameters, in particular, by the irradiation direction. The resulting NPSM with chiral nanoparticles inside can become a valuable substrate for the detection and investigation of natural chiral substances and for enhancement of the CPL of quantum objects.

## Data Availability

Data supporting results are available from the corresponding author upon reasonable request.

## Abbreviations

The following abbreviations are used in this manuscript:

CD	Circular dichroism
NPs	Nanoparticles
NPSM	Nanoporous silicate matrix
RHCL	Right-handed circular polarization
CPL	Circular polarized luminescence

## Appendix A

As can be seen from to the density spectra in Figure A3, we indicate the evidence incorporating gold NPSM into NPSM.

We also studied the influence of laser irradiation with right-handed circularly polarized light on the blank NPSM. It allows us to reveal the pure chiral contribution of 2D/3D gold NPs ensembles which was demonstrated in the main text (Figure 4).

In Figure A4, there is no substantial difference between the CD spectra of NPSM before and after laser irradiation. NPSM does not have a pronounced CD, but has a heterogeneity, which is explained by the heterogeneity of the pores inside the NPSM, as well as a great ability to scatter light. There is no doubt that the results shown in Figure 4 and Figure 5 are related to the anisotropy of gold NPs that occurs after laser irradiation.
